# Coronavirus infections in hospitalized pediatric patients with acute respiratory tract disease

**DOI:** 10.1186/1471-2334-12-365

**Published:** 2012-12-20

**Authors:** Monika Jevšnik, Tina Uršič, Nina Žigon, Lara Lusa, Uroš Krivec, Miroslav Petrovec

**Affiliations:** 1 Institute of Microbiology and Immunology, Faculty of Medicine, University of Ljubljana, Zaloška 4, Ljubljana 1000, Slovenia; 2 University Children’s Hospital, University Medical Centre Ljubljana, Bohoričeva 20, Ljubljana 1000, Slovenia; 3 Institute of Biostatistics and Medical Informatics, Faculty of Medicine, Vrazov trg 2, Ljubljana 1104, Slovenia

**Keywords:** Hospitalized children, Human coronavirus, Respiratory viral infection

## Abstract

**Background:**

Acute viral respiratory infections are an important cause of morbidity and mortality in humans worldwide. The etiological backgrounds of these infections remain unconfirmed in most clinical cases. The aim of this study was to estimate the prevalence of human coronavirus infections in a series of children hospitalized with symptoms of acute respiratory tract disease in a one-year period in Slovenia.

**Methods:**

The 664 specimens from 592 children under six years of age hospitalized at the University Children’s Hospital in Ljubljana were sent for the routine laboratory detection of respiratory viruses. Respiratory viruses were detected with a direct immunofluorescence assay and human coronaviruses were detected with a modified real-time RT–PCR.

**Results:**

HCoV RNA was detected in 40 (6%, 95% CI: 4.3%–8.1%) of 664 samples. Of these specimens, 21/40 (52.5%) were identified as species HKU1, 7/40 (17.5%) as OC43, 6/40 (15%) as 229E, and 6/40 (15%) as NL63. Infection with HCoV occurred as a coinfection with one or more other viruses in most samples (70%). Of the HCoV-positive children, 70.3% had lower respiratory tract infections.

**Conclusion:**

The results of our study show that HCoV are frequently detected human pathogens, often associated with other respiratory viruses and acute respiratory tract infections in hospitalized children. An association between age and the viral load was found. The highest viral load was detected in children approximately 10 months of age.

## Background

Acute infections of the respiratory tract are a major cause of morbidity and mortality in humans worldwide and approximately 80% are caused by viruses [1,2]. The viruses most frequently associated with respiratory tract infections include respiratory syncytial virus (RSV), parainfluenza viruses (PIV), influenza viruses (Flu), adenoviruses (AdV), human rhinoviruses (hRV), and enteroviruses, and less commonly, human metapneumovirus (hMPV), human bocavirus (HBoV), and human coronaviruses (HCoV). Until recently, it was believed that HCoVs were responsible for the common cold syndrome and were the cause of only mild upper respiratory tract infections (URTIs) [3]. After the SARS epidemic in 2003, it was established that these viruses can also be associated with severe, life-threatening, or even fatal respiratory infections [4]. Stewart at al. have also suggested a possible relationship between HCoV infections and the development of extrarespiratory symptoms, including some involving the central nervous system [5]. The role of HCoV in human gastrointestinal infections also awaits more detailed exploration [6,7].

The aim of this study was to estimate the prevalence of HCoV in a series of hospitalized infants and young children with symptoms of respiratory tract disease at the University Children’s Hospital in Ljubljana from June 2007 to May 2008. The main focus of the present study was to detect all four human coronaviruses in hospitalized children. A proportion of the patients included in this study have been partially described in a separate publication [8].

## Methods

### Study population

From June 2007 to May 2008, 897 respiratory specimens from 741 pediatric patients hospitalized at the University Children’s Hospital in Ljubljana with acute respiratory tract infections (ARTIs), were sent to the laboratory of the Institute of Microbiology and Immunology, Faculty of Medicine, University of Ljubljana, for the routine detection of respiratory viruses. The number of patients included in this study represented virtually all of hospitalized children with diagnosis of viral respiratory tract infection in this period.

Of these, 664 (74%) respiratory specimens (nasopharyngeal and throat swabs in viral transport medium, tracheal aspirates, bronchoalveolar lavage, or sputum) from 592 preschool children aged from three days to 72 months were included in to further analysis. Each of the 664 samples represented a different hospitalization event, except for one child from whom two different samples were taken and submitted for analysis (nasopharyngeal swab and bronchoalveolar lavage). Remaining 233 (26%) respiratory specimens were collected from 149 children either older than 72 months or were taken during the same hospitalization event. Therefore these specimens were excluded from the further analysis.

Demographic and clinical data were extracted from the children’s medical records and were available for 37 HCoV-positive children and 395 HCoV-negative children.

The present study was approved by the National Medical Ethics Committee, Ljubljana, Slovenia (no. 60/02/09).

### Laboratory investigations

Total nucleic acids were isolated from 190 μL of each respiratory specimen using a total nucleic acid isolation kit on a MagNA Pure Compact instrument (Roche Applied Science, Mannheim, Germany), according to the manufacturer’s instructions. An additional 5 μL of equine herpesvirus 1 and equine arteritis virus isolates were included in the nucleic acid extraction as internal controls and were detected in separate duplex PCR reactions with other specific targets [9,10].

The specimens were initially tested with direct immunofluorescence assays (DFAs; Oxoid, Cambridge, UK) for RSV, Flu A/B, PIV1-3, and AdV. After the routine detection assays, the remaining samples were stored frozen until their nucleic acid extraction.

All four human coronaviruses (229E, OC43, NL63, and HKU1) were detected with a modified coronavirus consensus real-time RT–PCR assay performed on a StepOne Real-Time PCR System (Applied Biosystems, Foster City, USA) to amplify 85–100-bp fragments of the polymerase 1b gene [11]. The real-time RT–PCR assays were performed as one-step reactions (SuperScript III Platinum One-Step Quantitative RT–PCR System; Invitrogen, CA, USA) within a single tube and a final volume of 20 μL, containing 5 μL of RNA and additional 6 mM MgSO4. The cycling conditions were as follows: 20 min at 50°C, 2 min at 95°C, and 45 cycles of 15 s at 95°C and 45 s at 60°C. All samples that were positive in the coronavirus screening assay were analyzed with a subtype-specific assay in which all the coronavirus species were detected separately [11]. All the specimens were also subsequently tested with real-time RT–PCR for the presence of hRV [12], hMPV [13], and HBoV [14] using previously published assays.

The relative quantity of viral RNA was estimated from the Ct values (cycle threshold; the cycle number at which the real-time fluorescent signal of the amplification target was above the background noise level) of the real-time PCR. A lower Ct value was interpreted as indicating a higher viral load.

### Statistical analysis

Categorical data are summarized as frequencies (%) and numerical data as medians (range).

The association between age and HCoV viral load was assessed with a linear regression model. Restricted cubic splines [15] were used to flexibly model the relationship between Ct in the HCoV assays and patient age. The shape of the estimated association is presented graphically [16].

We considered nine clinical characteristics (fever, oxygen support required, cough, rhinorrhea, dyspnea, wheezing, vomiting, location of the respiratory tract infection, and bronchiolitis) and used univariate logistic regression models with Firth’s correction to assess the association between HCoV positivity (covariate) and each clinical characteristic (response variables; these models are indicated as “models (a)” in the Results section). We further defined a three-category variable for the HCoV results: HCoV negative, HCoV positive with monoinfection, and HCoV positive with coinfection. We fitted an additional set of univariate models using the new variables as the covariates (“models (b)”). Only the first hospitalization for each child was considered in this analysis. The results are presented as odds ratios (OR) with 95% confidence intervals (95% CI) and *P* values.

The R statistical language was used for the analyses [16].

## Results

From June 2007 to May 2008, 664 specimens were collected from 592 children under six years of age with ARTI. The median age was 17.7 months (interquartile range: 9–28 months). The female: male ratio was 1:1.3 (256/592; 43% females). The 664 samples comprised 542 (81.5%) nasopharyngeal swabs, 102 (15.4%) throat swabs, and 20 (3%) other respiratory samples (i.e., tracheal aspirates).

### Virus detection

Overall, in 70.6% of samples, one or more viruses were confirmed with the combined results of RT–PCR and DFA. The prevalence of RSV, FluA, FluB, PIV1, PIV3, and AdV according to DFA was 12%, 0.1%, 0.1%, 1.2%, 0.6%, and 1%, respectively. PIV2 was not detected during the study. Human coronavirus RNA was detected in 40 (6%; 95% CI: 4.3%–8.1%) of the 664 samples, 33/40 (82.5%) in nasopharyngeal swab, 6/40 (15%) in throat swabs and 1/40 (2.5%) in bronchoalveolar lavage. Of the 40 specimens positive for coronavirus, 21/40 (52.5%) were HKU1, 7/40 (17.5%) were OC43, 6/40 (15%) were 229E, and 6/40 (15%) were NL63. All specimens were also tested with real-time RT–PCR for hRV, HBoV, and hMPV. The most common virus detected was hRV (37.6%), followed by HBoV (20.9%) and hMPV (11.3%).

**Figure 1:**
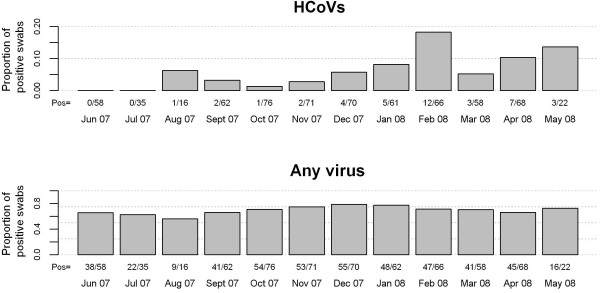
Seasonal distribution of positive results for coronavirus and other detected viruses.

Most HCoV infections were detected in winter (22/40, 55%) and spring (10/40, 25%), whereas 6/40 (15%) positive samples were collected in autumn and only 2/40 (5%) in summer. The association between HCoV positivity and seasonality was statistically significant (*P* < 0.001). February had the highest number of HCoV-positive specimens (12/40, 30%), whereas HCoV was not detected in June or July (Figure 1).

Of the 40 HCoV-positive samples, only 12 (30%) were shown to involve only one virus, including 3/6 229E, 2/7 OC43, 5/21 HKU1, and 1/6 NL63. Species 229E was most frequently detected as a monoinfection (50%), but one or more other viruses were identified in the majority (28/40, 70%) of coronavirus-positive samples: 21/40 (52.5%) were dual infections, 6/40 (15%) were triple infections, and four viruses were detected simultaneously in one infection (2.5%). The viruses most frequently detected with HCoV were hRV (42.8%), HBoV (32.1%), RSV (28.6%), hMPV (21.4%), and AdV (3.6%).

**Figure 2:**
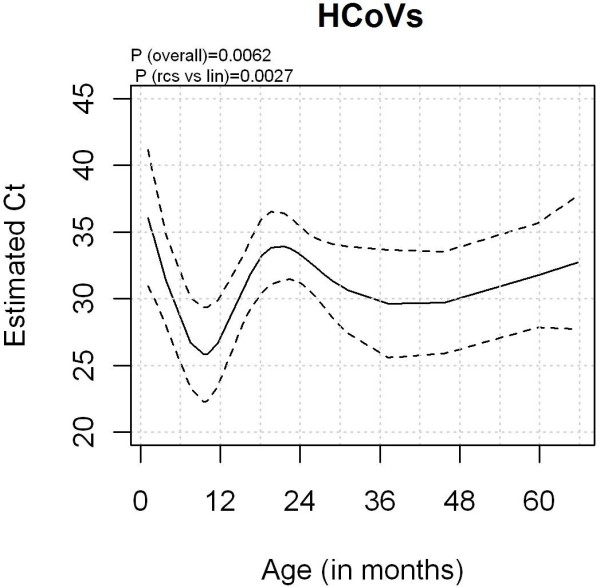
Estimated association between age and HCoV viral load (Ct).

On average, children with HCoV infections were older than those negative for HCoV (median ages in months: 23 vs 18 months, respectively), but infection with HCoV and age were not significantly associated (regardless of whether age was treated as a continuous or a categorical variable; data not shown). However, age was associated with the estimated quantity of HCoV viral nucleic acids present (measured as Ct). The association was nonlinear (*P* < 0.001) and the highest estimated viral load (lowest Ct) was detected in children around 10 months old. The estimated viral load increased with age for children younger than 10 months, whereas it decreased between 10 and 24 months. The estimated shape of the association is shown in Figure 2.

### Clinical features

Clinical data were available for 37 (92.5%) of the 40 HCoV-positive specimens and for 395/624 (63.3%) of the HCoV-negative specimens. The majority (70.3%) of HCoV-positive children were diagnosed with lower respiratory tract infections (LRTIs: bronchiolitis 56.7%, pneumonia 13.5%, bronchitis 5.4%, and recurrent wheezing 8.1%). The remaining patients (29.7%) had URTIs. Oxygen support during their hospital stay was required by 17/37 (45.9%) HCoV-positive children and the median duration was three days. The symptoms associated with HCoV are shown in Table 1.

**Table 1:** Demographic and clinical data for patients with and without human coronavirus infections

	**HCoV positive patients with characteristic**		**HCoV negative patients with characteristic**	
	**Number or median (n = 37)**	**% or range**	**Number or median (n = 395)**	**% or range**
Hospitalized	37	100	395	100
Median length of stay (range)	4	(2–17)	4	(1–20)
Fever	14	37.8	112	28
Oxygen support required	17	45.9	280	70.9
Median duration of oxygen support	3	(2–14)	3	(1–13)
Cough	23	62.2	311	78.7
Rhinorrhea	26	70.3	233	59
Conjunctivitis	1	2.7	16	4
Dyspnea	16	43.2	342	86.6
Wheezing	11	29.7	232	58.7
Vomiting	3	8.1	25	6.3
Diarrhea	1	2.7	7	1.8
Bronchitis	2	5.4	9	2.3
Bronchiolitis	21	56.7	349	88.3
Pneumonia	5	13.5	59	14.9
Recurrent wheezing	3	8.1	57	14.4
Asthma	2	8.7	22	5.6
Otitis media	1	2.7	14	3.5
Recurrent breathing/respiration difficulties	6	16.2	106	26.8
LRTIs	26	70.3	373	94.4

Key: *HCoV*, human coronavirus; *LRTIs*, lower respiratory tract infections.

The HCoV-positive children required significantly less oxygen support and had less cough, dyspnea, wheezing, LRTI, and bronchiolitis than HCoV-negative children (Table 2; models (a)). The differences in the clinical features of the HCoV-positive and HCoV-negative children were very similar to those observed between HCoV-positive children with monoinfections and HCoV-negative children (Table 2; models (b)), indicating that monoinfection with HCoV was associated with distinct clinical features. The only exception was the URTIs, which were more common among HCoV-positive children (28% for HCoV-positive children vs 5% for HCoV-negative children). Children with HCoV and coinfections had URTIs significantly more frequently than HCoV-negative children, and less frequently than HCoV-monoinfected children, the difference was statistically significant (P < 0.001) (4/10 = 40% among those with HCoV monoinfections, 6/26 = 23% among those with HCoV coinfections, and 18/373 = 5% among the HCoV-negative children).

**Table 2:** Odds ratios for the HCoV results according to the clinical characteristics

		**Models (a)**			**Models (b)**					
		**HCoV negative vs HCoV positive (n = 395 vs 37)**			**HCoV positive with coinfection vs HCoV positive with monoinfection (n = 26 vs 11)**			**HCoV negative vs HCoV positive with monoinfection (n = 395 vs 11)**		
		**OR**	**95% CI**	***P***	**OR**	**95% CI**	***P***	**OR**	**95% CI**	***P***
**Specific symptoms**	**Fever**	0.76	0.35,1.67	0.49	0.37	0.07,1.96	0.24	0.37	0.09,1.51	0.17
	**Oxygen support required**	2.46	1.18, 5.17	0.02	7.63	0.99,58.61	0.05	11.62	1.79,75.64	0.01
	**Cough**	2.03	0.93, 4.41	0.07	8.75	1.41,54.37	0.02	9.43	1.97,45.23	0.01
	**Rhinorrhea**	0.63	0.28,1.40	0.26	6.81	1.17,39.80	0.03	2.36	0.56,9.93	0.24
	**Dyspnea**	6.85	3.18,14.76	<0.001	7.63	0.99,58.61	0.05	32.26	4.90,213.37	<0.001
	**Wheezing**	2.79	1.28, 6.07	0.01	3.28	0.43,25.16	0.25	6.76	1.04, 43.92	0.05
	**Vomiting**	0.48	0.14, 1.61	0.23	2.90	0.11,74.05	0.52	0.99	0.05, 21.13	>0.99
**Diagnoses**	**LRTI vs URTI**	7.61	2.97,19.54	<0.001	2.62	0.44,15.54	0.29	15.72	3.26,75.81	<0.001
	**Bronchiolitis**	5.48	2.50,11.99	<0.001	5.72	0.96,33.94	0.05	19.58	4.03,95.12	<0.001

Key: The odds ratios and 95% CIs were derived from univariate logistic regression models. In models (a): the outcome is the presence of the specific symptom, HCoV positivity is the covariate, and the reference category is “HCoV positive”. In models (b), the outcome is the presence of the specific symptom, the covariate HCoV has three categories (HCoV positive with a monoinfection, HCoV positive with coinfection, and HCoV negative), and the reference category is “HCoV positive with monoinfection”.

An underlying medical condition was present in 11/37 (29.7%) HCoV infected patients, whereas the other HCoV-positive patients in this group were previously healthy. Four children with underlying disease carried HCoV as a single pathogen. Species 229E was more likely to be detected as a single infection than were the other viruses. Conversely, HKU1 was more likely to be detected as a coinfection with other viruses than as a monoinfection in children with an underlying medical condition. More than one virus was detected in 76.9% of HCoV-positive children without an underlying disease or immunodeficiency. HKU1 was detected as the only viral pathogen in a nasopharyngeal swab and bronchoalveolar lavage from a 9.7-month-old child with bronchiolitis who was previously healthy.

Because the number of cases in our study was small, no comparison of all the symptoms was made with a logistic regression model, nor was a comparison of the different coronavirus species and their associations with clinical symptoms possible. No differences were found between the C_T_ values of the HCoV-positive samples according to the different clinical outcomes (data not shown).

## Discussion

In our study, human coronavirus RNA was detected in 6% of respiratory samples from hospitalized children with ARTI. This prevalence was lower than we have found previously [8] and similar to that in other studies (from 2.6% to 8.7%) [17-20]. The majority of positive samples contained the HKU1 (52.5%) and the other coronavirus species were detected less frequently. A similar prevalence has been reported by Kuypers, and the prevalences of the HCoV species were reported in the same order [11]. There may be regional and annual variations in the circulation of different coronavirus species, as have been described by others [19]. HCoV was most often detected in winter and spring (80%), with the maximum number of cases presenting in February, whereas only 20% of samples were positive for HCoV in autumn and summer. Coronavirus infections have also been strongly associated with seasonality in previously published studies [20-23].

The children included in our study were under six years of age (median, 17.7 months). The probability of infection with HCoV was not significantly associated with age. However, the HCoV-infected patients in our study were older than those negative for HCoV. Some studies have included populations from all age groups [21,24,25], and coronaviruses have been detected in adults [26-29], young children [30-34], and neonates [35].

The association between age and the estimated viral load (from the Ct value) was interesting. The highest viral load was estimated for children about 10 months old (lower viral loads were detected in HCoV-infected children younger or older than 10 months). One reason for the highest viral load at this age may be the presence of maternal antibodies directed to HCoVs in younger infants [36].

The small number of cases and high number of HCoV coinfections with other respiratory viruses limited our ability to determine whether specific clinical signs or symptoms were associated with coronavirus infections. We compared only group of children infected with HCoV and those without HCoV infections. In our analysis children negative to all tested viruses were not included, because there is still uncertainty about possible infection with some of the newly described viruses (which were not included in our testing array). Children infected with HCoV required significantly less oxygen support, and had less cough, dyspnea, wheezing, LRTI, and bronchiolitis than the HCoV-negative children. URTIs were more frequent among HCoV-positive patients with monoinfections than among HCoV-negative children. As in other similar studies, we had only a relatively small number of coronavirus-positive samples. So far, only a few studies have included sufficient patients to permit a statistical comparison of the clinical outcomes of infections with different HCoV species [19,21,37]. It has recently been reported that coronavirus-infected children have LRTI less frequently than those infected with other viruses [38]. Children with coinfections had less severe disease than those with single virus infections. Children under 5 months of age had higher prevalence of single virus infections than older children [39]. In other recently published study the patients with coronavirus and influenza A virus coinfections had more severe disease than those with influenza virus infection only [40].

In the majority of our HCoV-positive specimens (70%), at least one other viral nucleic acid was present. To distinguish possible differences in the clinical presentations of children with a single infection from those of children with coinfections, and between the clinical presentations of infections with specific HCoV species, a study with more patients is required.

All HCoV-positive children included in our study were hospitalized with ARTI: 70.3% had LRTIs and 29.7% had URTIs. Pneumonia was diagnosed in 13.5% of children and 56.7% of children had bronchiolitis; 29.7% of HCoV-positive children had an underlying medical condition. As reported by others, single infections with HCoV were more often detected in children with an underlying medical condition than in previously healthy children: 5/11 (45.4%) vs 6/26 (23.1%), respectively [11]. In children with an underlying disease, the 229E was more often detected as a single infection than as a coinfection, whereas the HKU1 was more often detected as a coinfection.

Our study also contains several limitations. Due to the retrospective nature of the study, clinical data were not available for all included patients due to the noncomprehensive or missing clinical documentation. The reasons for the missing clinical data were that children were hospitalized due to the respiratory tract infection but were not stationed in pulmonary department and were distributed to other departments of the children's hospital because of the overcrowding. For these patients the clinical records were not available to investigators. Another limitation of this study is the fact that different samples were taken in some of the patients i.e. oropharyngeal instead of nasopharyngeal swab) and this could influence the yields of coronavirus (as well as other respiratory viruses). However due to the retrospective nature of the study we couldn't influence the sampling descisions made at the clinic. The politics of sample collection in place was that nasopharyngeal swab is preferred as the optimal sample but in cases where the physical or psychological conditions of the patient would not allow for safe nasopharyngeal swab collection, the laboratory would accept the oropharyngeal swab. The samples such as tracheal aspirates or BAL samples were collected from the severly affected children. All samples originated from hospitalized patients and that can also be considered as source of selection bias. Samples from outpatients and healthy control group children would be desirable in the study to determine HCoV prevalence and clinical features more accurately and also to exclude the possibility that viruses are detected as “contaminants” or “normal flora”. Due to the retrospective nature of the study, follow up testing was not performed. At the time of the study, detection of respiratory viruses was routinely performed by DFA instead of PCR which might underestimate the true prevalence of infections. However, we believe that these differences in sensitivity are not very prominent, especially in regard to detection of RSV virus and the fact that the study population represents young children in which DFA has best sensitivity.

## Conclusions

In conclusion, we found that all four non-SARS coronavirus were detected with ARTIs in hospitalized children in Slovenia and that their identification with routine diagnostic techniques is feasible. An association between age and the viral load was found. The highest viral load was detected in children approximately 10 months of age. Further investigation of HCoV, with the inclusion of a control group of healthy children, is required to better understand the clinical importance of HCoV in respiratory infections.

## Abbreviations

RSV: Respiratory syncytial virus; PIV: Parainfluenza viruses; Flu: Influenza viruses; AdV: Adenoviruses; hRV: Human rhinoviruses; hMPV: Human metapneumovirus; HBoV: Human bocavirus; HCoV: Human coronaviruses; URTIs: Upper respiratory tract infections; ARTIs: Acute respiratory tract infections; LRTIs: Lower respiratory tract infections.

## Competing interests

The authors declare that they have no competing interests.

This study was supported by the Slovenian Research Agency (Research Program P3-0083) and institutional department funds.

## Authors’ contributions

MJ and MP created the original idea of this research. MJ, TU, LL and MP designed the whole experiments and conducted analysis and interpretation of the data. NŽ, and UK participated in partial research work. All authors read and approved the final manuscript.

## Pre-publication history

The pre-publication history for this paper can be accessed here:

http://www.biomedcentral.com/1471-2334/12/365/prepub

## References

[B1] Bloom B Cohen RA Freeman G Summary health statistics for U.S. children: National Health Interview Survey, 2008. Vital and health Statistics 2009 244 1 81 20397379

[B2] Shay DK Holman RC Newman RD Liu LL Stout JW Anderson LJ Bronchiolitis-associated hospitalizations among US children, 1980–1996 JAMA 1999 282 15 1440 1446 10.1001/jama.282.15.1440 10535434

[B3] Mahony JB Detection of respiratory viruses by molecular methods Clin Microbiol Rev 2008 21 4 716 747 10.1128/CMR.00037-07 18854489 PMC2570148

[B4] Cheng VC Lau SK Woo PC Yuen KY Severe acute respiratory syndrome coronavirus as an agent of emerging and reemerging infection Clin Microbiol Rev 2007 20 4 660 694 10.1128/CMR.00023-07 17934078 PMC2176051

[B5] Stewart JN Mounir S Talbot PJ Human coronavirus gene expression in the brains of multiple sclerosis patients Virology 1992 191 1 502 505 10.1016/0042-6822(92)90220-J 1413524 PMC7131143

[B6] Esper F Ou Z Huang YT Human coronaviruses are uncommon in patients with gastrointestinal illness J Clin Virol 2010 48 2 131 133 10.1016/j.jcv.2010.03.007 20362494 PMC2864800

[B7] Risku M Lappalainen S Rasanen S Vesikari T Detection of human coronaviruses in children with acute gastroenteritis J Clin Virol 2010 48 1 27 30 10.1016/j.jcv.2010.02.013 20233673 PMC7108425

[B8] Ursic T Jevsnik M Zigon N Krivec U Beden AB Praprotnik M Petrovec M Human bocavirus and other respiratory viral infections in a 2-year cohort of hospitalized children J Med Virol 2012 84 1 99 108 10.1002/jmv.22217 22028039 PMC7167050

[B9] Diallo IS Hewitson G Wright L Rodwell BJ Corney BG Detection of equine herpesvirus type 1 using a real-time polymerase chain reaction J Virol Methods 2006 131 1 92 98 10.1016/j.jviromet.2005.07.010 16137772

[B10] Mankoc S Hostnik P Grom J Toplak I Klobucar I Kosec M Barlic-Maganja D Comparison of different molecular methods for assessment of equine arteritis virus (EAV) infection: a novel one-step MGB real-time RT-PCR assay, PCR-ELISA and classical RT-PCR for detection of highly diverse sequences of Slovenian EAV variants J Virol Methods 2007 146 1–2 341 354 17854913 10.1016/j.jviromet.2007.07.019

[B11] Kuypers J Martin ET Heugel J Wright N Morrow R Englund JA Clinical disease in children associated with newly described coronavirus subtypes Pediatrics 2007 119 1 e70 e76 10.1542/peds.2006-1406 17130280

[B12] Scheltinga SA Templeton KE Beersma MF Claas EC Diagnosis of human metapneumovirus and rhinovirus in patients with respiratory tract infections by an internally controlled multiplex real-time RNA PCR J Clin Virol 2005 33 4 306 311 10.1016/j.jcv.2004.08.021 15994117 PMC7185544

[B13] Maertzdorf J Wang CK Brown JB Quinto JD Chu M De Graaf M Van den Hoogen BG Spaete R Osterhaus AD Fouchier RA Real-time reverse transcriptase PCR assay for detection of human metapneumoviruses from all known genetic lineages Clin Microbiol Rev 2004 42 3 981 986 10.1128/JCM.42.3.981-986.2004 PMC356857 15004041

[B14] Lu X Chittaganpitch M Olsen SJ Mackay IM Sloots TP Fry AM Erdman DD Real-time PCR assays for detection of bocavirus in human specimens J Clin Microbiol 2006 44 9 3231 3235 10.1128/JCM.00889-06 16954253 PMC1594719

[B15] Harrell FE Jr Lee KL Pollock BG Regression models in clinical studies: determining relationships between predictors and response J Natl Cancer Inst 1988 80 15 1198 1202 10.1093/jnci/80.15.1198 3047407

[B16] RdC T R: A language and environment for statistical computing 2009 Vienna, Austria: R Development Core Team edn

[B17] Canducci F Debiaggi M Sampaolo M Marinozzi MC Berre S Terulla C Gargantini G Cambieri P Romero E Clementi M Two-year prospective study of single infections and co-infections by respiratory syncytial virus and viruses identified recently in infants with acute respiratory disease J Med Virol 2008 80 4 716 723 10.1002/jmv.21108 18297694 PMC7167101

[B18] Dominguez SR Robinson CC Holmes KV Detection of four human coronaviruses in respiratory infections in children: a one-year study in Colorado J Med Virol 2009 81 9 1597 1604 10.1002/jmv.21541 19626607 PMC2879166

[B19] Dare RK Fry AM Chittaganpitch M Sawanpanyalert P Olsen SJ Erdman DD Human coronavirus infections in rural Thailand: a comprehensive study using real-time reverse-transcription polymerase chain reaction assays J Infect Dis 2007 196 9 1321 1328 10.1086/521308 17922396 PMC7109921

[B20] Leung TF Li CY Lam WY Wong GW Cheuk E Ip M Ng PC Chan PK Epidemiology and clinical presentations of human coronavirus NL63 infections in hong kong children J Clin Microbiol 2009 47 11 3486 3492 10.1128/JCM.00832-09 19759228 PMC2772645

[B21] Lau SK Woo PC Yip CC Tse H Tsoi HW Cheng VC Lee P Tang BS Cheung CH Lee RA Coronavirus HKU1 and other coronavirus infections in Hong Kong J Clin Microbiol 2006 44 6 2063 2071 10.1128/JCM.02614-05 16757599 PMC1489438

[B22] Chiu SS Chan KH Chu KW Kwan SW Guan Y Poon LL Peiris JS Human coronavirus NL63 infection and other coronavirus infections in children hospitalized with acute respiratory disease in Hong Kong, China Clin Infect Dis 2005 40 12 1721 1729 10.1086/430301 15909257 PMC7107956

[B23] Bastien N Anderson K Hart L Van Caeseele P Brandt K Milley D Hatchette T Weiss EC Li Y Human coronavirus NL63 infection in Canada J Infect Dis 2005 191 4 503 506 10.1086/426869 15655772 PMC7199484

[B24] Arden KE Nissen MD Sloots TP Mackay IM New human coronavirus, HCoV-NL63, associated with severe lower respiratory tract disease in Australia J Med Virol 2005 75 3 455 462 10.1002/jmv.20288 15648064 PMC7166768

[B25] Lee BE Robinson JL Khurana V Pang XL Preiksaitis JK Fox JD Enhanced identification of viral and atypical bacterial pathogens in lower respiratory tract samples with nucleic acid amplification tests J Med Virol 2006 78 5 702 710 10.1002/jmv.20595 16555283 PMC7166532

[B26] Woo PC Lau SK Tsoi HW Huang Y Poon RW Chu CM Lee RA Luk WK Wong GK Wong BH Clinical and molecular epidemiological features of coronavirus HKU1-associated community-acquired pneumonia J Infect Dis 2005 192 11 1898 1907 10.1086/497151 16267760 PMC7110183

[B27] Kistler A Avila PC Rouskin S Wang D Ward T Yagi S Schnurr D Ganem D DeRisi JL Boushey HA Pan-viral screening of respiratory tract infections in adults with and without asthma reveals unexpected human coronavirus and human rhinovirus diversity J Infect Dis 2007 196 6 817 825 10.1086/520816 17703411 PMC7109683

[B28] Garbino J Crespo S Aubert JD Rochat T Ninet B Deffernez C Wunderli W Pache JC Soccal PM Kaiser L A prospective hospital-based study of the clinical impact of non-severe acute respiratory syndrome (Non-SARS)-related human coronavirus infection Clin Infect Dis 2006 43 8 1009 1015 10.1086/507898 16983613 PMC7107919

[B29] Brittain-Long R Westin J Olofsson S Lindh M Andersson LM Prospective evaluation of a novel multiplex real-time PCR assay for detection of fifteen respiratory pathogens-duration of symptoms significantly affects detection rate J Clin Virol 2010 47 3 263 267 10.1016/j.jcv.2009.12.010 20080440 PMC7108433

[B30] Lambert SB Allen KM Druce JD Birch CJ Mackay IM Carlin JB Carapetis JR Sloots TP Nissen MD Nolan TM Community epidemiology of human metapneumovirus, human coronavirus NL63, and other respiratory viruses in healthy preschool-aged children using parent-collected specimens Pediatrics 2007 120 4 e929 e937 10.1542/peds.2006-3703 17875651

[B31] Esposito S Bosis S Niesters HG Tremolati E Begliatti E Rognoni A Tagliabue C Principi N Osterhaus AD Impact of human coronavirus infections in otherwise healthy children who attended an emergency department J Med Virol 2006 78 12 1609 1615 10.1002/jmv.20745 17063525 PMC7166434

[B32] Fabbiani M Terrosi C Martorelli B Valentini M Bernini L Cellesi C Cusi MG Epidemiological and clinical study of viral respiratory tract infections in children from Italy J Med Virol 2009 81 4 750 756 10.1002/jmv.21457 19235872 PMC7167005

[B33] Van der Hoek L Ihorst G Sure K Vabret A Dijkman R De Vries M Forster J Berkhout B Uberla K Burden of disease due to human coronavirus NL63 infections and periodicity of infection J Clin Virol 2010 48 2 104 108 10.1016/j.jcv.2010.02.023 20347384 PMC7108429

[B34] Pierangeli A Gentile M Di Marco P Pagnotti P Scagnolari C Trombetti S Lo Russo L Tromba V Moretti C Midulla F Detection and typing by molecular techniques of respiratory viruses in children hospitalized for acute respiratory infection in Rome, Italy J Med Virol 2007 79 4 463 468 10.1002/jmv.20832 17311326 PMC7166338

[B35] Gagneur A Dirson E Audebert S Vallet S Legrand-Quillien MC Laurent Y Collet M Sizun J Oger E Payan C Materno-fetal transmission of human coronaviruses: a prospective pilot study Eur J Clin Microbiol Infect Dis 2008 27 9 863 866 10.1007/s10096-008-0505-7 18373106 PMC7087967

[B36] Dijkman R Jebbink MF Gaunt E Rossen JW Templeton KE Kuijpers TW Van der Hoek L The dominance of human coronavirus OC43 and NL63 infections in infants J Clin Virol 2012 53 2 135 139 10.1016/j.jcv.2011.11.011 22188723 PMC7108278

[B37] Vabret A Dina J Gouarin S Petitjean J Tripey V Brouard J Freymuth F Human (non-severe acute respiratory syndrome) coronavirus infections in hospitalised children in France J Paediatr Child Health 2008 44 4 176 181 10.1111/j.1440-1754.2007.01246.x 17999671 PMC7166728

[B38] Kristoffersen AW Nordbo SA Rognlien AG Christensen A Dollner H Coronavirus causes lower respiratory tract infections less frequently than RSV in hospitalized Norwegian children Pediatr Infect Dis J 2011 30 4 279 283 10.1097/INF.0b013e3181fcb159 21057374

[B39] Martin ET Kuypers J Wald A Englund JA Multiple versus single virus respiratory infections: viral load and clinical disease severity in hospitalized children Influenza Other Respi Viruses 2012 6 1 71 77 10.1111/j.1750-2659.2011.00265.x 21668660 PMC3175338

[B40] Esper FP Spahlinger T Zhou L Rate and influence of respiratory virus co-infection on pandemic (H1N1) influenza disease J Infect 2011 63 4 260 266 10.1016/j.jinf.2011.04.004 21546090 PMC3153592

